# Levosimendan in critically ill adults: a utilisation review

**DOI:** 10.1186/cc12164

**Published:** 2013-03-19

**Authors:** J Aron, S Harrison, A Milne, S Patel, R Maharaj

**Affiliations:** 1 Lewisham NHS Trust, London, UK; 2 King's College Hospital NHS Trust, London, UK

## Introduction

Levosimendan improves haemodynamic performance and may have cardioprotective effects. Small trials have demonstrated efficacy in well-defined populations [1]. Given the uncertain benefits we describe contemporaneous patient selection, utilisation of levosimedan, haemodynamic effects and outcomes.

## Methods

The study was performed at a single centre's three ICUs. Data from 38 consecutive admissions, from 2010 to 2012, treated with levosimendan were collected retrospectively. Demographics, illness severity, comorbidity, haemodynamic, metabolic, biochemical, resource utilisation, organ support and hospital outcomes were analysed.

## Results

Our cohort had a mean age of 58 (95% CI = 53.4 to 64.4). Only 18% underwent cardiothoracic surgery: sepsis, pulmonary emboli and myocardial/pericardial diseases were also treated with levosimendan. Admission characteristics included mean PaO_2_/FiO_2 _ratio (PFR) of 186.7 mmHg (95% CI = 160 to 212) and mean cardiac index (CI) of 2.3 (95% CI 1.9 to 2.8). The median APACHE II score was 24 (interquartile range 18.5 to 30). Levosimendan was initiated at a variable point during ICU treatment (days 0 to 14) and was usually the third inotrope (range 0 to 5) commenced. Treatment with levosimendan (Table 1) resulted in improved CI and PFR with reductions in EVLW, BE, lactate and creatinine. The ICU length of stay was 15.4 days (95% CI = 13.2 to 17.6) and the hospital mortality was 54%. No significant adverse effects were reported.

**Table 1:** Treatment outcomes

Variable	Mean	95% CI
CI Δd5 - d0	+1.34	0.4 to 2.2
PFR Δd5 - d0	+284	223 to 345
BE Δd5 - d0	+2.9	0.85 to 5
Lac Δd5 - d0	-1	-2 to 0.2

d0, treatment commenced.

## Conclusion

Levosimendan is usually used in patients with cardiogenic shock unresponsive to conventional inotropes or mechanical augmentation. The mortality of this group is high but represents patients with shock refractory to conventional treatment. Its use in sepsis, myocarditis and pulmonary embolism is not well established. In this group levosimendan appears to have a favourable effect on gas exchange, renal function and tissue perfusion. Limitations include retrospective analysis and missing data.
